# Identifying Associations in Minimum Inhibitory Concentration Values of *Escherichia coli* Samples Obtained From Weaned Dairy Heifers in California Using Bayesian Network Analysis

**DOI:** 10.3389/fvets.2022.771841

**Published:** 2022-04-27

**Authors:** Brittany L. Morgan, Sarah Depenbrock, Beatriz Martínez-López

**Affiliations:** ^1^Public Health Sciences, School of Medicine, University of California, Davis, Davis, CA, United States; ^2^Center for Animal Disease Modeling and Surveillance, Department of Veterinary Medicine and Epidemiology, School of Veterinary Medicine, University of California, Davis, Davis, CA, United States; ^3^Department of Veterinary Medicine and Epidemiology, School of Veterinary Medicine, University of California, Davis, Davis, CA, United States

**Keywords:** bovine, weaned, enteric, Bayesian network analysis, Bayesian, minimum inhibitory concentration, antibiotics

## Abstract

**Objective:**

Many antimicrobial resistance (AMR) studies in both human and veterinary medicine use traditional statistical methods that consider one bacteria and one antibiotic match at a time. A more robust analysis of AMR patterns in groups of animals is needed to improve on traditional methods examining antibiotic resistance profiles, the associations between the patterns of resistance or reduced susceptibility for all isolates in an investigation. The use of Bayesian network analysis can identify associations between distributions; this investigation seeks to add to the growing body of AMR pattern research by using Bayesian networks to identify relationships between susceptibility patterns in *Escherichia coli* (*E. coli*) isolates obtained from weaned dairy heifers in California.

**Methods:**

A retrospective data analysis was performed using data from rectal swab samples collected from 341 weaned dairy heifers on six farms in California and selectively cultured for *E. coli*. Antibiotic susceptibility tests for 281 isolates against 15 antibiotics were included. Bayesian networks were used to identify joint patterns of reduced susceptibility, defined as an increasing trend in the minimum inhibitory concentration (MIC) values. The analysis involved learning the network structure, identifying the best fitting graphical mode, and learning the parameters in the final model to quantify joint probabilities.

**Results:**

The graph identified that as susceptibility to one antibiotic decreases, so does susceptibility to other antibiotics in the same or similar class. The following antibiotics were connected in the final graphical model: ampicillin was connected to ceftiofur; spectinomycin was connected with trimethoprim-sulfamethoxazole, and this association was mediated by farm; florfenicol was connected with tetracycline.

**Conclusions:**

Bayesian network analysis can elucidate complex relationships between MIC patterns. MIC values may be associated within and between drug classes, and some associations may be correlated with farm of sample origin. Treating MICs as discretized variables and testing for joint associations in trends may overcome common research problems surrounding the lack of clinical breakpoints.

## Introduction

Antimicrobial resistance (AMR) is a complex phenomenon and one of the biggest public health challenges of our time (1). AMR is a naturally occurring process, but is accelerated when the presence of evolutionary pressures, such as antibiotics, pressure bacteria to adapt (2). Such evolutionary pressures are present in hospitals, communities, farms, and the natural environment, making AMR an issue that spans multiple sectors that truly requires a One Health approach (3, 4). Many efforts are made to understand risk factors for AMR in human and animal medicine. However, to accurately identify risk factors and areas for intervention, the outcome needs to be carefully considered. Bacteria often exhibit complex patterns of resistance to different antibiotic drugs. This complexity makes risk factor analyses for AMR potentially challenging (5).

Multiple resistance patterns are common and develop from a complex system comprised of both biological and evolutionary mechanisms (6). These mechanisms are interconnected with many of the normal processes existing in the livestock production sector, such as antibiotic use, biosecurity and farm hygiene, farm husbandry practices, and livestock flow between farms. To identify risk factors for resistance and explore the potential reservoirs of plasmid-associated resistance genes of public health significance on farms, we must improve our understanding of the current state of AMR in livestock animals. However, many efforts made toward identifying and understanding AMR in the agricultural sector (7–9) consider only one bacteria and one antibiotic match at a time. While this is suitable for some investigations, it is arguably an oversimplification and fails to describe antibiotic resistant profiles, or the associations between the patterns of resistance or reduced susceptibility for all isolates in an investigation. Increasingly, studies are exploring resistance profiles by grouping several antibiotics and using whether a bacteria is simultaneously resistant to the grouped antibiotics as the outcome of interest. Bayesian networks can build on the efforts by describing the complex web of resistance profiles and identify associations between individual patterns.

One of the most widely used approaches in epidemiological analyses, and the current standard in AMR centered research, remains multivariable regression modeling (10). While this method identifies statistical associations, it assumes the predictors, or independent variables, are not correlated with each other. However, antibiotic drugs may be multicollinear if they are in the same drug class, spectrum, or target bacteria in similar ways. Multidrug resistance in bacteria is more akin to observing multivariate observations, where there are correlations between the individual resistance patterns (11). Implementing multivariate analyses in AMR studies offers a richer modeling framework and can provide a greater understanding of disease process (10). Bayesian networks are graphical models of the relationships among a set of random variables (12, 13). Using this form of statistical modeling, we can infer a probabilistic model which describes a joint probability structure (11). Previous studies have used Bayesian network analysis to elucidate complex, statistical dependent relationships (14–17), including some specifically looking at AMR patterns in the agriculture sector (11, 18, 19). However, there is limited evidence for using Bayesian network analysis to analyze minimum inhibitory concentrations (MIC) in ways not requiring a qualitative interpretation.

Interpretive standards for MIC classification of “resistant” or “susceptible” require breakpoints, which are the MIC value that delineates antimicrobial susceptibility categories (resistant vs. susceptible) for a specific bacterium and antibiotic combination. These breakpoints are established by the Clinical and Laboratory Standards Institute (CLSI) (20) and use knowledge of pharmacokinetic data for the drug in question in reference to the site of infection, are specific for the species in which the infection exists, and are thus designed to describe specific host/pathogen/antibiotic relationships to predict likelihood of a bacteriologic cure within that described relationship. Because these CLSI breakpoints apply to specific host/pathogen/antibiotic relationships, applicable breakpoints are not available for every scenario in which an MIC may be investigated. There are no CLSI breakpoints for the interpretation of enteric bacterial MICs isolated from the feces of cattle. This leads clinicians or researchers to either extrapolate from related host/pathogen/antibiotic breakpoints or use wild-type epidemiological cut-off values (21). Reporting and analyzing quantitative MIC data, rather than dichotomizing the results and analyzing qualitative data, provides a mechanism to detect shifts in MIC trends over time, facilitate early detection of reducing susceptibility (22), compare data, and more thoroughly explore relationships between antibiotics. Further, these methods are not subject to changes in clinical breakpoints.

Using Bayesian network analysis to model the joint patterns of reduced susceptibility from within the livestock production environment permits a more complete understanding of the epidemiology of AMR. The present study seeks to add to this growing body of research by evaluating whether there are patterns of reduced susceptibility in *Escherichia coli (E. coli)* across different antibiotics. Using an observational retrospective study design, we conducted a Bayesian network analysis to identify patterns using MIC values for 15 antibiotics for *E. coli* isolates obtained from weaned dairy heifer fecal samples in California. We hypothesized MIC values for antibiotics belonging to the same class would be jointly associated. As this hypothesis is well-established, seeing linkages between these drugs will provide evidence supporting our proposed method for testing joint associations without interpretive breakpoints.

## Materials and Methods

### Data Source and Sampling Protocol

This study was conducted as a secondary analysis using existing culture and sensitivity data from a previous investigation of respiratory and enteric bacterial MICs in weaned dairy heifers <6 months of age in California (23). These data represent a total of 341 weaned dairy heifers, sampled from mixed weaned pens from six California calf rearing operations, and include samples from calves both with and without signs of bovine respiratory disease (BRD) based on a validated scoring system for weaned heifers (24). Sampling was conducted at two different seasonal time points (spring/summer and fall/winter) for each of the six facilities. Rectal swabs were collected from weaned dairy heifers in group pens and <6 months of age. Swabs were refrigerated at four degrees Celsius for no more than 2 days until a batch could be shipped to the study laboratory at the California Animal Health and Food Safety laboratory located in Davis, CA for selective culture and sensitivity. The data analysis reported herein used only existing culture and sensitivity data from a previous study conducted with IACUC approval; no additional animals were used for this secondary analysis.

### Antimicrobial Susceptibility Analyses

Samples obtained via rectal swabs were selectively cultured for *E. coli*. Isolates were tested for antimicrobial susceptibility using broth microdilution (Trek Sensititre, Trek Diagnostic Systems, Thermo Fisher Scientific, Waltham, MA) according to CLSI guidelines (25) to determine the MIC of the 19 antimicrobial drugs contained on the Sensititre Bovine BOPO7F Vet AST plate (Thermo Scientific, Remel Inc., Lenexa, KS, USA). The microbroth dilution plates contained the following antimicrobials: ceftiofur (CEF), penicillin (PEN), ampicillin (AMP), tiamulin (TIA), tylosin (TYL), tulathromycin (TUL), tilmicosin (TILM), clindamycin (CLN), tildipirosin (TILD), tetracycline (TET), gentamicin (GEN), neomycin (NEO), gamithromycin (GAM), florfenicol (FLR), danofloxacin (DAN), enrofloxacin (ENR) sulphadimethoxine (SUL), trimethoprim-sulfamethoxazole (SXT), and spectinomycin (SPC).

### Bayesian Network Analysis

One farm was removed from the study analysis due to missing animal records data. This left a total of five farms and 281 *E. coli* samples in the analysis, one isolate per animal included. Minimum inhibitory concentration values were categorized to guarantee at least 10 observations per category. If an MIC value had <10 observations, it was regrouped with the lower MIC value to be conservative. If the lowest MIC value had <10 observations, it was grouped with the next higher value. MIC values were treated as discretized variables and used to identify joint patterns of reduced susceptibility using Bayesian network analysis. Reduced susceptibility was defined as a trend of increasing MIC value (26). The antibiotics PEN, CLN, TILM, and TYL were excluded from the analysis due to all cultured bacteria having the maximum MIC value tested. The remaining 15 antibiotic MICs were maintained for analysis.

Non-MIC variables collected across farms and without missing data were also incorporated in the analysis. These included the season the sample was collected, the age of the heifer in days, categorized by quartile, and the farm from which the sample was taken to account for clustering. There are three distinct parts to our network analysis, as previously described: (27) (i) learn the network structure -i.e., the relationship, connections or arcs between the nodes- and identify the best fitting model; (ii) learn the parameters included in the final selected model; and (iii) bootstrap analysis. All analyses were conducted in R using the Bayesian network learning package “bnlearn” (28).

We used a purely data-driven, exploratory approach to model the relationships between our set of variables. We did not make prior assumptions of causal relationships (i.e., forcing paths) or restrict paths between variables. The constraint-based PC algorithm identifies the optimal directed acyclic graph, a single graph that best captures the joint dependencies between the variables in the data set (29). Constraint-based algorithms identify conditional independence with statistical tests and link nodes (variables) that are found to be non-independent (30). We chose the constraint-based PC algorithm because constraint-based algorithms are more accurate than score-based algorithms for small sample sizes (30). Further, constraint-based algorithms allowed us to incorporate a statistical test capable of evaluating independence for non-binary variables.

Choosing the conditional independence test to use depends on the distribution of variables in the network. For discrete networks, log-likelihood ratio tests are the most commonly used, Gaussian mutual information for Gaussian networks, and Fisher's *Z* test and exact *t*-test are often used for partial correlation (30). Due to a lack of breakpoints, we were unable to categorize the MIC values to an interpretation of *resistant* or *susceptible*. Further, MICs are not continuous variables as they can be both left- and right-censored. MIC values cannot be treated as discretized variables because there is an innate ordering between the intervals. The inability to categorize the MICs, as well as the observed censoring, limits our choice of statistical test. The Jonckheere-Terpstra (JT) test provides a non-parametric alternative to evaluate joint associations between ordered variables. The JT test is a rank-based test used to determine if there are upward or downward monotonic trends in the data (29). We chose this test to explore resistance, or in this case MIC, profiles when lacking clinical breakpoints.

### Bootstrap Analyses

To identify which arcs were most consistent and strongest, a bootstrapping approach was used (27). This method uses the optimal model from the constraint-based PC search described in the previous section, 3.1.3, to generate 10,000 bootstrap samples using the *boot.strength* function. For each bootstrap sample, the previous steps are repeated, and a separate network is learned using the constraint-based PC learning algorithm and the JT test. The *boot.strength* function returns the strength of connection for each pair of nodes (i.e., how frequently the connection is observed). This information is used to build a consensus network, defined as a network containing arcs having a strength >50% (11, 31, 32). Arcs observed in more than 50% of the bootstrapped samples are included in an averaged, consensus network using the *averaged.network* function (27). This averaged, consensus model then incorporates the information on the strength of the connections by weighting the arcs. The averaged, consensus model is then visually compared to the optimal model identified in Bayesian Network Analysis.

### Parameter Learning

After identifying the averaged, consensus model, we used the *bn.fit* function to model the parameters. Parameter learning estimates the conditional probabilities between connected nodes from the previously identified network and the observed sample data (33). We used the Bayesian method for parameter learning because we had no missing data. Also, this method uses the expected values of the parameters' posterior distribution arising from a flat prior and is less prone to overfitting in Bayesian parameter estimation than maximum likelihood estimation. Using *cpquery* we were able to perform conditional probability queries and estimate the probability of an association between two nodes or events. The objective of this study was to identify the statistical dependencies between observed *E. coli* susceptibility, measured as MIC, to several antibiotics. Additionally, given the cross-sectional nature of the data, arc direction was not relevant and we present the graphical model with undirected connections for simplicity of interpretation (2).

## Results

### Descriptive Statistics

**Figure 1:**
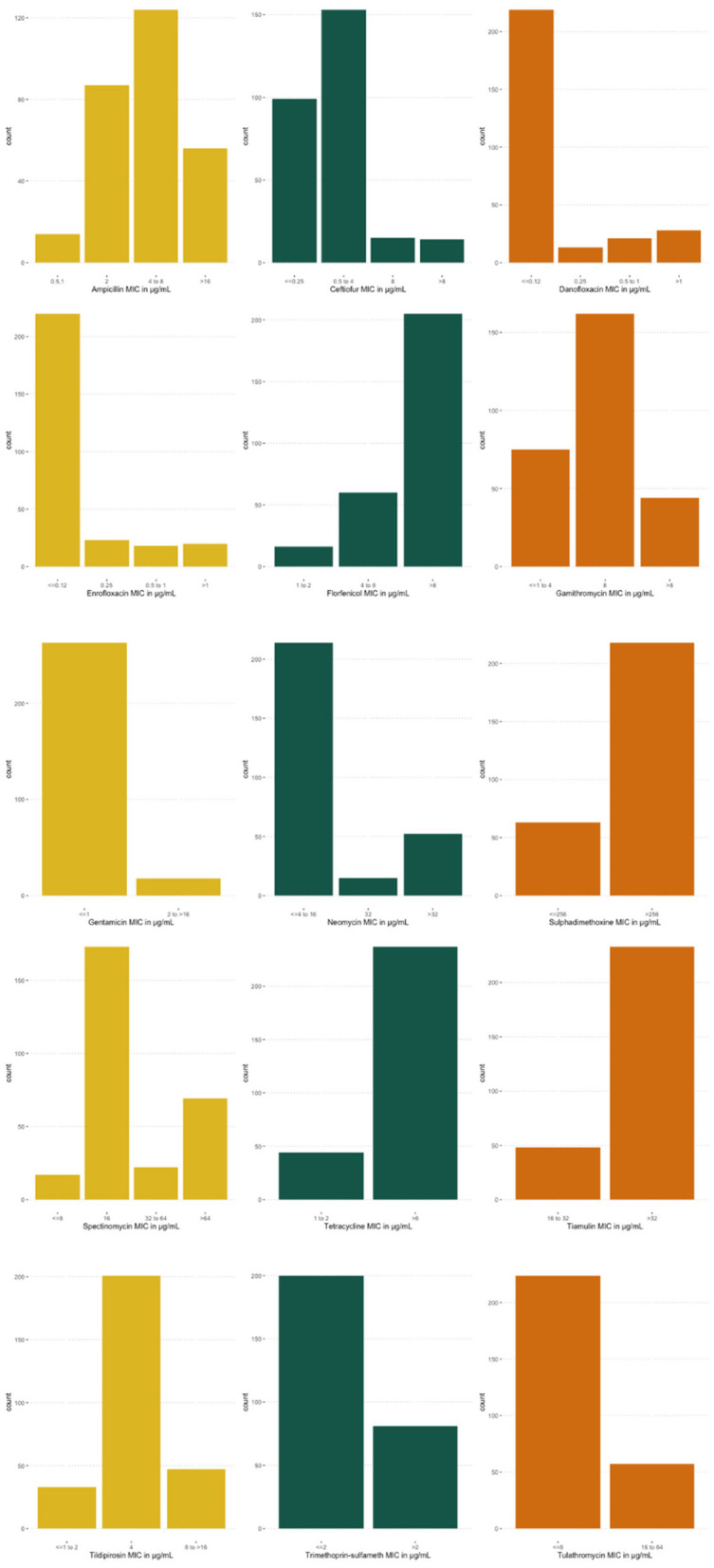
Distributions of minimum inhibitory concentrations (MIC) for *Escherichia coli* (*E. coli*) isolates. MIC distribution for *E. coli* isolates, collected from five farms for 15 antibiotics, analyzed in Bayesian network analysis.

Data from two-hundred and eighty-one calves were included in the study. Of those, 98 were Jersey, 179 were Holstein, and 4 were a Jersey/Holstein crossbreed. The average age of heifer was 132 days (95% CI: 129, 134). Fifty-six (±3) isolates were collected and tested from each farm. MIC value distributions for antibiotics included in the analysis can be seen in Figure 1. Most *E. coli* isolates were inhibited at low concentrations of CEF, GEN, NEO, SXT, and TUL. MIC values were highest for FLR, SUL, TET, and TIA. Descriptive statistics for all variables in the dataset are represented in Table 1.

**Table 1:** Descriptive statistics of isolates included in sample.

**Descriptive**	**No. isolates (%)**
**Sampled farm**	
1	53 (18.9)
2	59 (21.0)
3	54 (19.2)
4	58 (20.6)
5	57 (20.3)
**Season sampled**	
Summer	139 (49.5)
Winter	142 (50.5)
**Sampled breed**	
Jersey	98 (34.9)
Holstein	179 (63.7)
Jersey/Holstein	4 (1.4)
**Age of calf sampled (days)**	
Mean, 95% CI	132 (129, 134)

*Summary of isolates obtained from weaned heifers sampled across five farms in California*.

### Model Results

**Figure 2:**
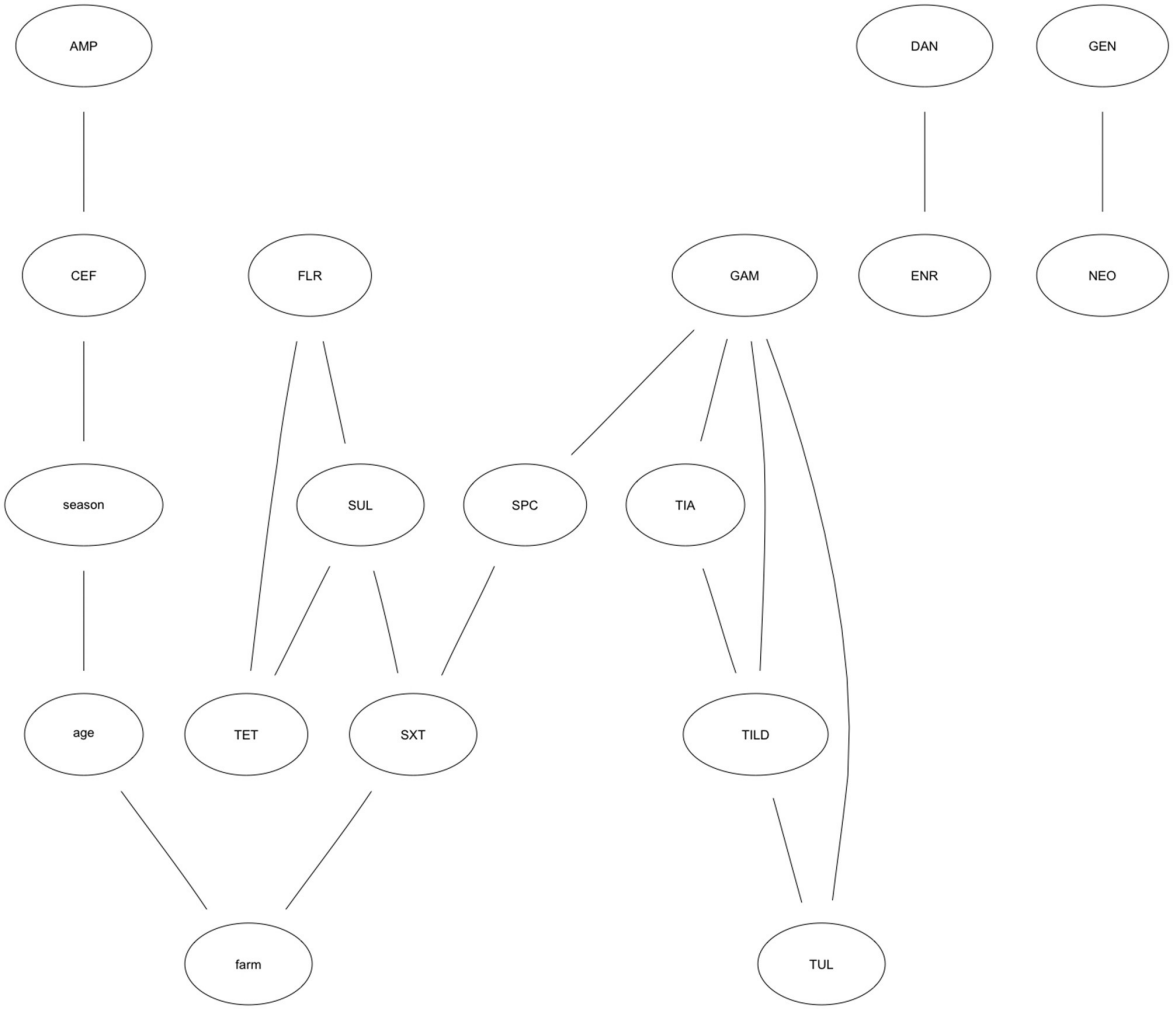
Optimal Bayesian network model showing relationships among antibiotic MIC trends. Optimal Bayesian network modeling MIC patterns for *Escherichia coli* isolates collected from weaned heifers across five farms in California against 15 antibiotics. Antibiotics represented in each node include: CEF, Ceftiofur; AMP, Ampicillin; TIA, Tiamulin; TUL, Tulathromycin; TILD, Tildipirosin; TET, Tetracycline; GEN, Gentamicin; NEO, Neomycin; GAM, Gamithromycin; FLR, Florfenicol; DAN, Danofloxacin; ENR, Enrofloxacin; SUL, Sulphadimethoxine; SXT, Trimethoprim-Sulfamethoxazole; SPC, Spectinomycin.

The results of the optimal network is presented graphically. Figure 2 shows the resulting graph, which contained 18 nodes and 18 arcs. The following antibiotic characteristics are linked together: susceptibility to AMP is positively associated with susceptibility to CEF, indicating as MIC values for one antibiotic increase, MIC values for the other also increase. SPC is positively associated with GAM. The probability an isolate has the maximum MIC value for SPC (>64 μg/mL), given it has the maximum MIC value for GAM (>8 μg/mL) is 76%. The association is also positive in the reverse order GAM—SPC, however it is not as strong (43%). FLR—TET are inversely associated, indicating MIC values for one antibiotic increase as they decrease for the other. Other connections indicating joint associations in MIC trend include SXT—SUL, TET—SUL, SUL—FLR, SPC—SXT, TIA—TILD, TIA—GAM, TILD—TUL, TILD—GAM, TUL—GAM, DAN—ENR, GEN—NEO.

TET—SUL—FLR and TIA—TILD—GAM—TUL form joint motifs of susceptibility. That is, arcs jointly connect these nodes and indicate a complex relationship between these antibiotics. The probability an isolate has an MIC value for SUL >256 μg/mL given the MIC value for TET was >8 μg/mL is 89%. Conversely, the probability an isolate has an MIC value for TET >8 μg/mL given the MIC value for SUL is >256 μg/mL is 95%. The probability an isolate has the highest MIC value for SUL given the highest MIC value for TET is higher when the MIC value for FLR is >8 μg/mL (93%) than when FLR is 1–2 μg/mL (<0.01%). The probability an isolate has the highest MIC value for both TUL and TILD (TUL—TILD = 58%; TILD—TUL = 47%) differs based on the MIC for GAM. When GAM is >8 μg/mL, the probability an isolate has the highest MIC values for TUL—TILD is 81%, compared to <0.01% when GAM is ≤ 1 to 4 μg/mL. The effect of GAM holds true for TILD—TUL, also. The probability an isolate has an MIC for TIA >32 μg/mL given an MIC for GAM >8 μg/mL is 84% without the influence of TILD. Taking TILD into account, the probability an isolate has a high MIC for TIA given it has a high MIC for GAM and TILD (8 to >16 μg/mL) is 98%.

While DAN—ENR and GEN—NEO are connected, their relationship is completely independent of any other antibiotics in the analysis as indicated by their separation from the other nodes in the graph. Further, SXT is the only node with a direct relationship to farm; the probability that an isolate has the maximum MIC value for SXT was 49% in one farm. However, for another farm the probability for this same relationship was 5%. While potential risk factors were not included in this analysis, farm level management practices, characteristics of the herd at each farm, or treatment history could explain the differences in the probability an isolate has an MIC for SXT >2 μg/mL between farms.

### Bootstrap Analyses

**Figure 3:**
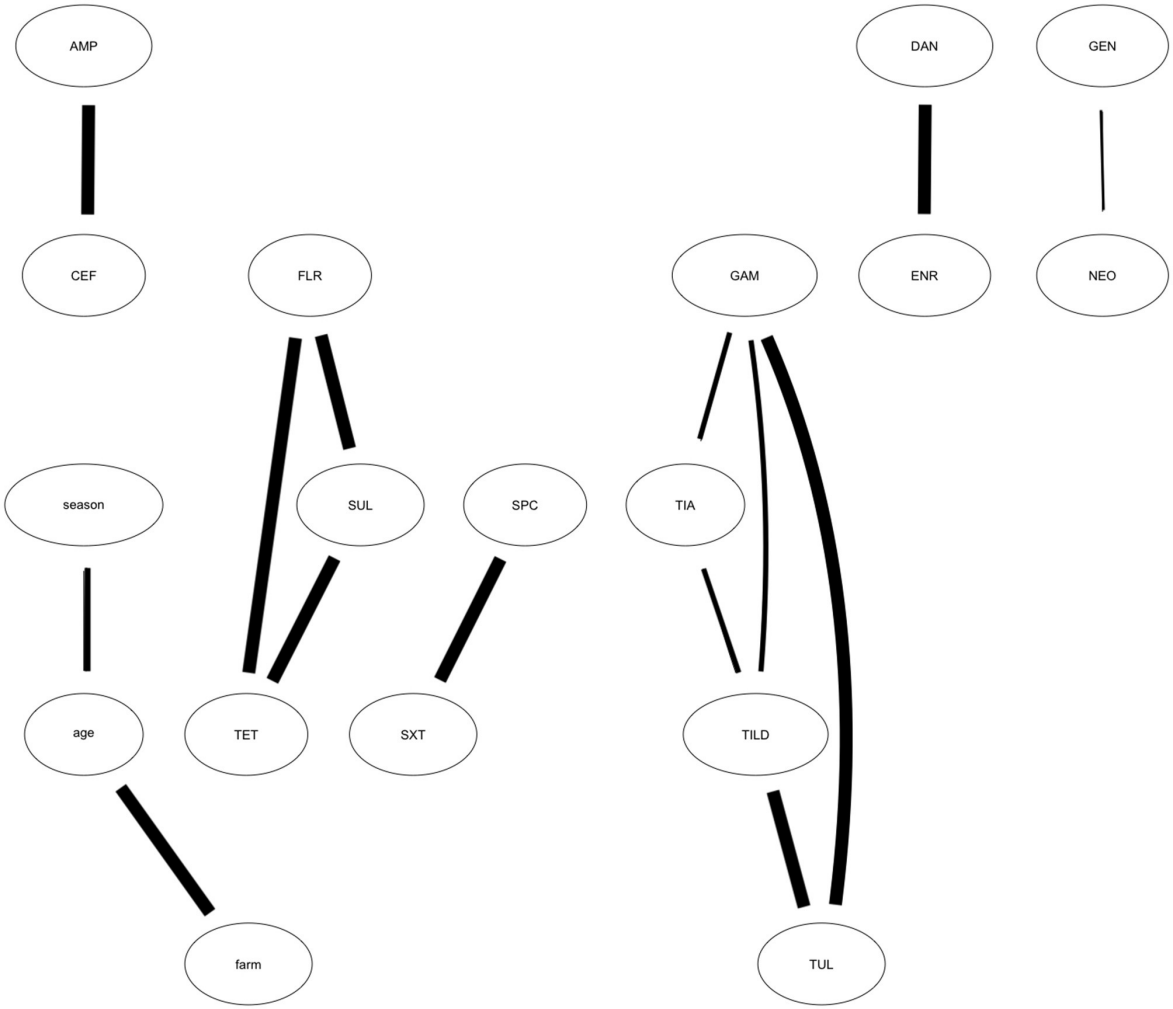
Averaged, Consensus network model showing results of bootstrapped analysis. Averaged, consensus network developed from the optimal network showing the relationships among antibiotic MIC trends. The averaged, consensus network depicts the arcs identified from the optimal network that appear in more than 50% of the 10,000 bootstrapped samples and are most strongly supported by the data. Strength of connection is denoted by arc weight (i.e., the thicker the arc, the greater strength). Antibiotics represented in each node include: CEF, Ceftiofur; AMP, Ampicillin; TIA, Tiamulin; TUL, Tulathromycin; TILD, Tildipirosin; TET, Tetracycline; GEN, Gentamicin; NEO, Neomycin; GAM, Gamithromycin; FLR, Florfenicol; DAN, Danofloxacin; ENR, Enrofloxacin; SUL, Sulphadimethoxine; SXT, Trimethoprim-Sulfamethoxazole; SPC, Spectinomycin.

Using 10,000 bootstrap samples, we identified MIC relationships more robustly supported. From the bootstrap searches, we identified 14 of the 18 arcs met the strength threshold of 50%. These arcs were used to build the averaged, consensus network which included AMP—CEF, DAN—ENR, TET—FLR, TET—SUL, TIA—TILD, TIA—GAM, TILD—GAM, TUL—GAM, GEN—NEO, SPC—SXT, season—age, farm—age. The connections not maintained in the averaged, consensus network, and therefore only loosely supported, included CEF—season, SUL—SXT, SXT—farm, and SPC—GAM. The arcs most strongly supported are identified by those with the thickest lines connecting the nodes (Figure 3). These include AMP—CEF, DAN—ENR, TET—FLR—SUL, TIA—TILD—TUL—GAM, SPC—SXT, and Farm—Age. Conditional probabilities of the highest and lowest MIC value for the MIC associations maintained in the bootstrap analysis are presented in Table 2.

**Table 2:** Conditional probabilities for MIC associations maintained in the bootstrapped analysis.

MIC associations^*^	**Probability**
P (AMP: >16 μg/mL | CEF: >8 μg/mL)	99%
P (AMP: >16 μg/mL | CEF: ≤ 0.25 μg/mL)	6%
P (AMP: 0.5–1 μg/mL | CEF: ≤ 0.25 μg/mL)	99%
P (DAN: >1 μg/mL | ENR: >1 μg/mL)	99%
P (DAN: >1 μg/mL | ENR: ≤ 0.12 μg/mL)	<1%
P (DAN: ≤ 0.12 μg/mL | ENR: ≤ 0.12 μg/mL)	99%
P (TET: >8 μg/mL | SUL: >256 μg/mL)	95%
P (TET: >8 μg/mL | SUL: ≤ 256 μg/mL)	46%
P (TET: 1–2 μg/mL | SUL: ≤ 256 μg/mL)	57%
P (TET: >8 μg/mL | FLR: >8 μg/mL)	98%
P (TET: >8 μg/mL | FLR: 1–2 μg/mL)	7%
P (TET: 1–2 μg/mL | FLR: 1–2 μg/mL)	93%
P (TIA: >32 μg/mL | GAM: >8 μg/mL)	84%
P (TIA: >32 μg/mL | GAM: ≤ 1–4 μg/mL)	64%
P (TIA: 16–32 μg/mL | GAM: ≤ 1–4 μg/mL)	37%
P (TIA: 2 >32 μg/mL | TILD: 8 to >16 μg/mL)	97%
P (TIA: >32 μg/mL | TILD: ≤ 1 to 2 μg/mL)	43%
P (TIA: 16–32 μg/mL | TILD: ≤ 1 to 2 μg/mL)	59%
P (TUL: 16–64 μg/mL | TILD: 8 to >16 μg/mL)	58%
P (TUL: 16–64 μg/mL | TILD: ≤ 1 to 2 μg/mL)	10%
P (TUL: ≤ 8 μg/mL | TILD: ≤ 1 to 2 μg/mL)	91%
P (TUL: 16–64 μg/mL | GAM: >8 μg/mL)	51%
P (TUL: 16–64 μg/mL | GAM: ≤ 1 to 4 μg/mL)	3%
P (TUL: ≤ 8 μg/mL | GAM: ≤ 1 to 4 μg/mL)	96%
P (TILD: 8 to >16 μg/mL | GAM: >8 μg/mL)	26%
P (TILD: 8 to >16 μg/mL | GAM: ≤ 1 to 4 μg/mL)	2%
P (TILD: ≤ 1 to 2 μg/mL | GAM: ≤ 1 to 4 μg/mL)	29%
P (GEN: 2 to >16 μg/mL | NEO: >32 μg/mL)	20%
P (GEN: 2 to >16 μg/mL | NEO: ≤ 4 to 16 μg/mL)	3%
P (GEN: ≤ 1 μg/mL | NEO: ≤ 4 to 16 μg/mL)	97%
P (SPC: >64 μg/mL | SXT: >2 μg/mL)	63%
P (SPC: >64 μg/mL | SXT: ≤ 2 μg/mL)	11%
P (SPC: ≤ 8 μg/mL | SXT: ≤ 2 μg/mL)	8%

* *Antibiotics: AMP, Ampicillin; CEF, Ceftiofur; DAN, Danofloxacin; ENR, Enrofloxacin; TET, Tetracycline; SUL, Sulphadimethoxine; FLR, Florfenicol; TIA, Tiamulin; GAM, Gamithromycin; TILD, Tildipirosin; TUL, Tulathromycin; GEN, Gentamicin; NEO, Neomycin; SXT, Trimethoprim-Sulfamethoxazole; SPC, Spectinomycin*.

*Select conditional probabilities for MIC variables from the bootstrapped Bayesian network model. Associations presented below represent the most consistent associations (i.e., strongest) in the optimal model, as identified by the bootstrap analysis. That is, they appear in more than 50% of the bootstrap replicates. Probability that Escherichia coli MIC for one antibiotic is true given that the MIC for the corresponding antibiotic is true. MIC values for each antibiotic chosen as highest categorized value or lowest categorized value*.

## Discussion

Not surprisingly, in the graphic model generated through Bayesian network analysis, antibiotics belonging to the same classes are linked together. GAM, TIA, TUL, and TILD are linked and are all macrolides. Other linkages according to class include beta-lactams AMP and CEF; sulfonamides SXT and SUL; aminoglycosides GEN and NEO; and quinolones DAN and ENR. These connections indicate that the MIC value to one antibiotic in a class is associated with the MIC value to other antibiotics in that class. This is not surprising because antibiotics in the same class have similar mechanisms of action, and thus bacterial resistance mechanisms are often effective against more than one antibiotic in a class depending on the mechanism of resistance (34). However, it is important to note a linkage in the graph does not denote causality. An antibiotic connected to another antibiotic implies a systematic dependency in trend for the observed MIC values (11). For example, TIA, TILD, TUL, and GAM are all connected in the graphical model indicating they are mutually dependent and should be investigated jointly in any risk factor analysis searching for causal determinants (11). Further, antibiotics of different classes may play a role in selection pressure on *E. coli* populations. For instance, ceftiofur resistance has been found on calf farms despite low ceftiofur use (35). There is evidence demonstrating florfenicol treatment in dairy calves drives the coselection of florfenicol- and ceftiofur-resistance in *E.coli* (36). While other studies have suggested ceftiofur-resistance is more closely related to the practice of feeding calves waste milk, potentially due to drug residues (37–39). We did not observe a relationship between FLR and CEF. However, we did not test for joint associations in resistance. Instead, we focused on associations among trends in MIC values. We were not able to incorporate information regarding waste milk practices due to all enrolled farms from which these data were obtained feeding waste milk. We did observe relationships between other MIC variables from antibiotics of different classes that could be indicative of coselection.

FLR and TET are connected in the graph; these antibiotics are part of the different phenicol and tetracycline classes. A recent study in swine demonstrated that tylosin exposure reduced susceptibility of *Salmonella* to both drugs (40). Bacteria exposure to sub-lethal doses of tylosin acquired resistance mechanisms to the antibiotic. Exposure to tylosin also resulted in activation of the efflux system and its global regulators, which subsequently increased the MICs of FLR and TET against *Salmonella* (40). This finding may explain why FLR and TET are connected in our model, however recent tylosin exposure was not reported in the study population. The MIC values for tylosin were not included in our analysis because all bacteria cultured had the maximum MIC value for the drug. Because all isolates had reduced susceptibility to tylosin, it is possible the relationship between FLR, TET, and tylosin could also hold true for other non-*Salmonella* enteric pathogens, such as *E. coli*. Future studies should explore the role tylosin treatment has in the coselection of flurofenicol- and tetracycline-resistance in *E. coli* populations among cattle.

Our graph identified an association between TET and SUL, and this relationship was maintained in the bootstrap analysis. These antibiotics belong to two different, but important classes of drugs used in calves. High percentages of TET and SUL resistance in calves has been reported elsewhere (41). Sulfanomide, tetracycline, and aminoglycoside is a common resistance pattern for *E. coli* in dairy calves (41–43). Our results loosely support the association between these antibiotic classes. Arcs are present between SPC, SXT, SUL, and TET. SXT and SUL are both sulfanomides, TET is a tetraycline, and SPC is an amiocyclitol, which is an antibiotic class that shares several similarities with aminoglycosides. However, it is interesting SXT—SUL was not an arc retained in the bootstrap analysis, since they are of the same class, and instead SXT—SPC was separated from TET—FLR—SUL.

The farm from which the sample was collected was directly connected to only SXT and this connection was not maintained in the bootstrap analysis. Originally, we believed including the farm variable could represent unmeasured farm-level risk factors that may be associated with MIC values. It is possible there was too much variation in an all-encompassing farm variable to identify systematic associations between farm characteristics and MIC values. We were unable to incorporate specific farm-level risk factors that could influence the joint distributions in MIC values, such as antibiotic use and route of administration or farm management and husbandry practices due to the level of missingness in these measurements. However, these relationships were investigated in the larger study from which these data were obtained. Finally, we excluded penicillin, clindamycin, and tilmicosin from the analysis as all isolates had the maximum MIC value. This could be due to natural resistance in *E. coli* to these substances as other studies have demonstrated 100% resistance to these drugs (44, 45).

## Limitations

Our use of MIC values due to the lack of clinically relevant breakpoints for commensal *E. coli* in dairy cattle inhibits our ability to distinguish if an isolate is “resistant” or “susceptible.” However, using MIC values rather than interpretations allowed us to make inferences on profiles and relationships without extrapolating or adapting breakpoints from other organisms or hosts. The small sample size combined with the geographic location from which isolates were collected, limits generalizability of the findings beyond California. However, the methods employed can be incorporated into future, larger studies seeking to identify AMR profiles and risk factors for AMR. Additionally, we did not include farm-level variables in this analysis, which limits our ability to explore the risk factors or drivers of the observed associations. This work is being conducted in the larger, original study from which this data set was obtained. Finally, there is no known or validated model to compare our optimal model with. Thus, we expect some of the arcs represent spurious associations. Our bootstrap analysis retained 14 connections, representing the associations in which we have a higher degree of confidence are non-spurious. The ability to link our results to biology and confirm the structure of our model in the absence of a validated model would strengthen our findings. This could be done by conducting genomic analysis and identifying genes coding for resistance that may better explain the conditional dependencies observed in this data analysis.

## Conclusions

It is not surprising to observe associations among susceptibility to antibiotics of the same class because they often have similar mechanisms of action. However, we identified MIC trends linked outside of antibiotic class. These findings are clinically important because they suggest that AMR in enteric organisms is interrelated and use patterns may convey less easily predicable patterns of resistance to drugs not recently used on farm. Thus, changing use patterns, or attempts to decrease AMR in enteric organisms, will not likely be straight forward; removing the use of one drug may not decrease AMR to that drug without also removing the use of other drugs to which that AMR pattern is associated, or removing other non-antimicrobial influences on AMR. While the isolates in this study are commensal fecal *E. coli*, and do not necessarily represent disease causing agents, the information is valuable because it elucidates relationships between isolates, and not just the relationship between an individual isolate and a single antibiotic. The linkages demonstrated in this data analysis that are beyond antibiotic class are hypothesis generating; they suggest investigation into why certain drugs may be linked is warranted, and that AMR control efforts should consider the more complicated associations of conditional susceptibilities between related and unrelated drugs and animal management factors.

Animal, farm, and environmental level characteristics may have an impact on antibiotic associations, which is an important area for future research. Many studies identify associations between farm variables and resistance, but few describe risk factor variables and how they affect the relationships among MICs. As previously mentioned, it is common to evaluate the risk factors for one bacteria and one antibiotic match at a time (7–9, 46–50). Considering the relationships between different antibiotics, with the antibiotic susceptibility profiles, will create a more accurate model of the interactions that likely affect AMR. Future research could include similar analyses for additional bacteria species in the same biologic niches, both individually and in conjunction with other species, because it is possible that bacteria inhabiting the same hosts or niches influence each other (51). Further, work directed at identifying risk factors on the animal, farm, and environmental level will provide a more thorough understanding of AMR complexity by explaining the occurrence of susceptibility or resistance profiles. Finally, conducting genomic analyses and identifying genetic elements, and variations in expression that lead to resistance could link our and future modeling results to biologic outcomes.

Our study adds to the growing body of research exhibiting how Bayesian network analysis can elucidate complex relationships between MIC patterns. We demonstrate how treating MICs as discretized variables and testing for joint associations in trends in MIC values may overcome common research problems surrounding the lack of clinical breakpoints.

## Data Availability Statement

The data analyzed in this study is subject to the following licenses/restrictions: a simplified, anonymous version of the dataset used in this study is included in the Supplementary Materials. The original dataset is part of a veterinary medical record and cannot be made publicly available. Requests to access these datasets should be directed to blmorgan@ucdavis.edu.

## Author Contributions

BM led conceptualization and analysis of the project. SD was principal investigator on the original study and provided the data as well as consultation during the analysis. BM-L provided consultation during the data analysis process. All authors participated in the writing and editing of the submitted article and have given their final approval.

## Funding

The original study from which these data were collected was provided by the California Department of Food Agriculture.

## Conflict of Interest

The authors declare that the research was conducted in the absence of any commercial or financial relationships that could be construed as a potential conflict of interest.

## Publisher's Note

All claims expressed in this article are solely those of the authors and do not necessarily represent those of their affiliated organizations, or those of the publisher, the editors and the reviewers. Any product that may be evaluated in this article, or claim that may be made by its manufacturer, is not guaranteed or endorsed by the publisher.
