# Supplementary material for: Identifying Associations in Minimum Inhibitory Concentration Values of Escherichia coli Samples Obtained From Weaned Dairy Heifers in California Using Bayesian Network Analysis
Source: Front Vet Sci. 2022 Apr 27;9:771841. doi: 10.3389/fvets.2022.771841 (PMC9093072; doi:10.3389/fvets.2022.771841)
Supplement: Supplementary file 1 [file Data_Sheet_1.PDF]

Analysis of Minimum Inhibitory Concentration Values for Escherichia coli in Morgan et al., Frontiers (2022)

Brittany L Morgan

1/25/2022

The data

The raw data contains 281 isolates with 19 minimum inhibatory concentration (MIC) variables. Four of the variables were removed for analysis due to all isolates having the maximum MIC value, leaving the following variables:

1. AMP: ampicillin
2. CEF: ceftiofur
3. DAN: danofloxacin
4. ENR: enrofloxacin
5. FLR: florfenicol
6. GAM: gamithromycin
7. GEN: gentamicin
8. NEO: neomycin
9. SUL: sulphadimethoxine
10. SPC: spectinomycin
11. TET: tetracycline
12. TIA: tiamulin
13. TILD: tildipirosin
14. SXT: trimethoprim-sulfamethoxazole
15. TUL: tulathromycin

Libraries Needed

```
library(bnlearn)
library(ggplot2)
library(ggpubr)
library(dplyr)
```

```
##
## Attaching package: 'dplyr'
```

```
## The following objects are masked from 'package:stats':
##
##     filter, lag
```

```
## The following objects are masked from 'package:base':
##
##     intersect, setdiff, setequal, union
```

The data can be loaded from ecoli-mic.csv (link).

```
ecoli.mic=read.csv("ecoli-mic.csv")
```

Preprocessing and exploratory data analysis

Specifcy the variables are ordinal with the given levels and apply MIC value labels

```
ecoli.ordinal <- ecoli.mic

ecoli.ordinal$AMP <- factor(ecoli.mic$AMP, order = TRUE,
                          levels = c(1, 2, 3, 4),
                          labels = c("0.5 to 1", "2", "4 to 5", ">16"))
ecoli.ordinal$CEF <- factor(ecoli.mic$CEF, order = TRUE,
                           levels = c(1, 2, 3, 4),
                           labels = c("<=0.25", "0.5 to 4", "8", ">8"))
ecoli.ordinal$DAN <- factor(ecoli.mic$DAN, order = TRUE,
                            levels = c(1, 2, 3, 4),
                            labels = c("<=0.12", "0.25", "0.5 to 1", ">1"))
ecoli.ordinal$ENR <- factor(ecoli.mic$ENR, order = TRUE,
                            levels = c(1, 2, 3, 4),
                            labels = c("<=0.12", "0.25", "0.5 to 1", ">1"))
ecoli.ordinal$FLR <- factor(ecoli.mic$FLR, order = TRUE,
                           levels = c(1, 2, 3),
                           labels = c("1 to 2", "4 to 8", ">8"))
ecoli.ordinal$GAM <- factor(ecoli.mic$GAM, order = TRUE,
                           levels = c(1, 2, 3),
                           labels = c("<=1 to 4", "8", ">8"))
ecoli.ordinal$GEN <- factor(ecoli.mic$GEN, order = TRUE,
                           levels = c(1, 2),
                           labels = c("<=1", "2 to >16"))
ecoli.ordinal$NEO <- factor(ecoli.mic$NEO, order = TRUE,
                            levels = c(1, 2, 3),
                            labels = c("<=4 to 16", "32", ">32"))
ecoli.ordinal$SUL <- factor(ecoli.mic$SUL, order = TRUE,
                            levels = c(1, 2),
                            labels = c("<=256", ">256"))
ecoli.ordinal$SPC <- factor(ecoli.mic$SPC, order = TRUE,
                            levels = c(1, 2, 3, 4),
                            labels = c("<=8", "16", "32 to 64", ">64"))
ecoli.ordinal$TET <- factor(ecoli.mic$TET, order = TRUE,
                            levels = c(1, 2),
                            labels = c("1 to 2", ">8"))
ecoli.ordinal$TIA <- factor(ecoli.mic$TIA, order = TRUE,
                            levels = c(1, 2),
                            labels = c("16 to 32", ">32"))
ecoli.ordinal$TILD <- factor(ecoli.mic$TILD, order = TRUE,
                            levels = c(1, 2, 3),
                            labels = c("<=1 to 2", "4", "8 to >16"))
ecoli.ordinal$SXT <- factor(ecoli.mic$SXT, order = TRUE,
                            levels = c(1, 2),
                            labels = c("<=2", ">2"))
ecoli.ordinal$TUL <- factor(ecoli.mic$TUL, order = TRUE,
                            levels = c(1, 2),
                            labels = c("<=8", "16 to 64"))

summary(ecoli.ordinal)
```

```
##        AMP           CEF           DAN           ENR          FLR
##  0.5 to 1: 14   <=0.25  : 99   <=0.12  :219   <=0.12  :220   1 to 2: 16
##  2       : 87   0.5 to 4:153   0.25    : 13   0.25    : 23   4 to 8: 60
##  4 to 5  :124   8       : 15   0.5 to 1: 21   0.5 to 1: 18   >8    :205
##  >16     : 56   >8      : 14   >1      : 28   >1      : 20
##        GAM           GEN            NEO          SUL          SPC
##  <=1 to 4: 75   <=1     :263   <=4 to 16:214   <=256: 63   <=8     : 17
##  8       :162   2 to >16: 18   32       : 15   >256 :218   16      :173
##  >8      : 44                  >32      : 52               32 to 64: 22
##                                                            >64     : 69
##      TET           TIA           TILD      SXT          TUL
##  1 to 2: 44   16 to 32: 48   <=1 to 2: 33   <=2:200   <=8     :224
##  >8    :237   >32     :233   4       :201   >2 : 81   16 to 64: 57
##                              8 to >16: 47
##
```

Explore the MIC distributions

```
amp <- ggplot(ecoli.ordinal, aes(AMP)) + xlab("Ampicillin MIC in μg/mL") + geom_bar(fill
= "#dbb522") + theme_pubclean() +
      scale_x_discrete(breaks=c("1","2","3", "4"),labels=c("0.5,1", "2", "4 to 8", ">16"
))
cef <- ggplot(ecoli.ordinal, aes(CEF)) + xlab("Ceftiofur MIC in μg/mL") + geom_bar(fill
 = "#155547") + theme_pubclean() +
      scale_x_discrete(breaks=c("1","2","3", "4"),labels=c("<=0.25", "0.5 to 4", "8", ">
8"))
dan <- ggplot(ecoli.ordinal, aes(DAN)) + xlab("Danofloxacin MIC in μg/mL") + geom_bar(fi
ll = "#ce6b11") + theme_pubclean() +
      scale_x_discrete(breaks=c("1","2","3", "4"),labels=c("<=0.12", "0.25", "0.5 to 1",
">1"))
enr <- ggplot(ecoli.ordinal, aes(ENR)) + xlab("Enrofloxacin MIC in μg/mL") + geom_bar(fi
ll = "#dbb522") + theme_pubclean() +
      scale_x_discrete(breaks=c("1","2","3", "4"),labels=c("<=0.12", "0.25", "0.5 to 1",
">1"))
flr <- ggplot(ecoli.ordinal, aes(FLR)) + xlab("Florfenicol MIC in μg/mL") + geom_bar(fil
l = "#155547") + theme_pubclean() +
      scale_x_discrete(breaks=c("1","2","3"),labels=c("1 to 2", "4 to 8", ">8"))
gam <- ggplot(ecoli.ordinal, aes(GAM)) + xlab("Gamithromycin MIC in μg/mL")+ geom_bar(fi
ll = "#ce6b11") + theme_pubclean() +
      scale_x_discrete(breaks=c("1","2","3"),labels=c("<=1 to 4", "8", ">8"))
ggarrange(amp, cef, dan, enr, flr, gam, ncol = 3, nrow = 2)
```

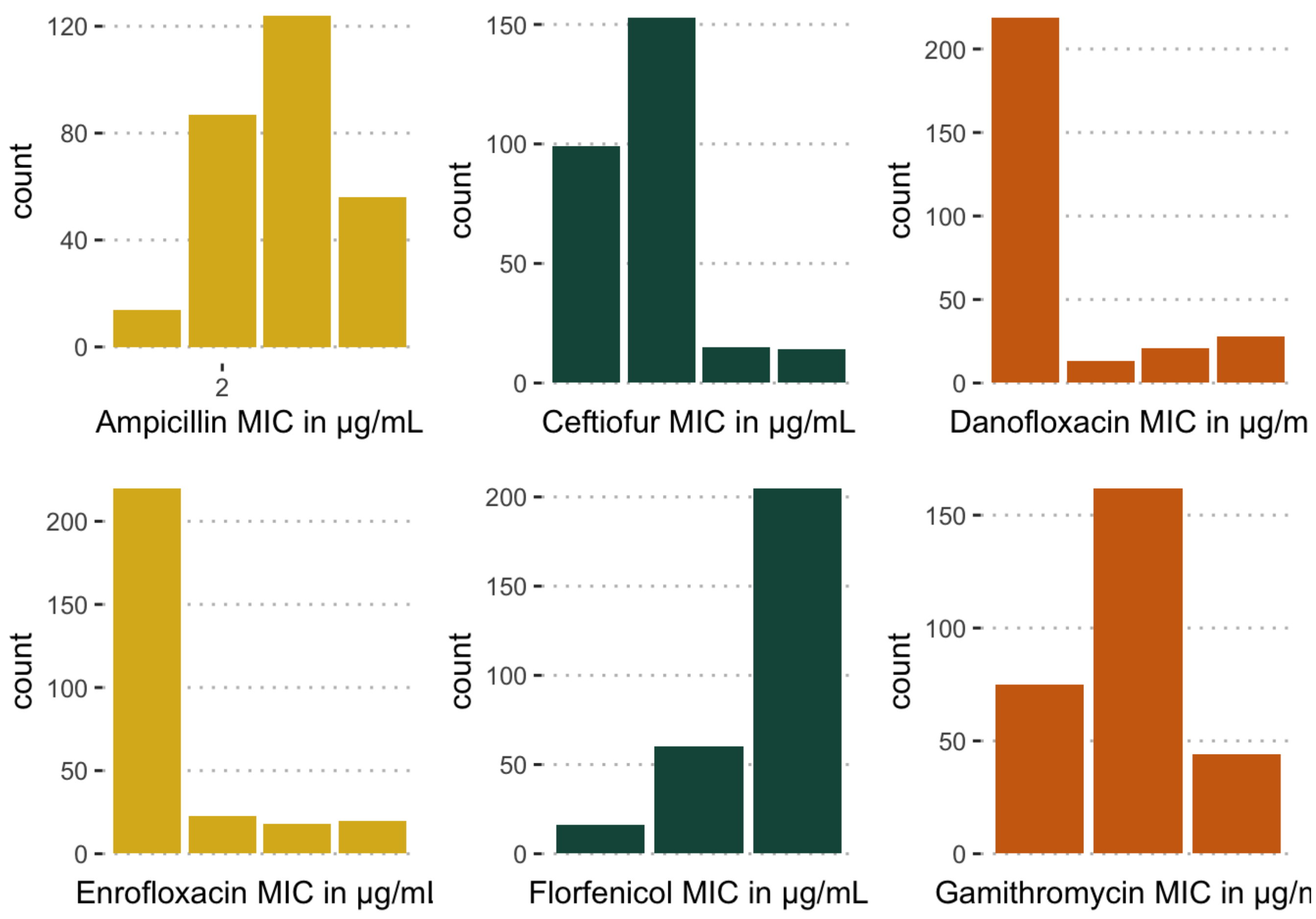


```
gen <- ggplot(ecoli.ordinal, aes(GEN)) + xlab("Gentamicin MIC in µg/mL") + geom_bar(fill
= "#dbb522") + theme_pubclean() +
      scale_x_discrete(breaks=c("1","2"),labels=c("<=1", "2 to >16"))
neo <- ggplot(ecoli.ordinal, aes(NEO)) + xlab("Neomycin MIC in µg/mL") + geom_bar(fill =
"#155547") + theme_pubclean() +
      scale_x_discrete(breaks=c("1","2","3"),labels=c("<=4 to 16", "32", ">32"))
sul <- ggplot(ecoli.ordinal, aes(SUL)) + xlab("Sulphadimethoxine MIC in µg/mL") + geom_b
ar(fill = "#ce6b11") + theme_pubclean() +
      scale_x_discrete(breaks=c("1","2"),labels=c("<=256", ">256"))
spc <- ggplot(ecoli.ordinal, aes(SPC)) + xlab("Spectinomycin MIC in µg/mL") + geom_bar(f
ill = "#dbb522") + theme_pubclean() +
      scale_x_discrete(breaks=c("1","2","3", "4"),labels=c("<=8", "16", "32 to 64", ">6
4"))
tet <- ggplot(ecoli.ordinal, aes(TET)) + xlab("Tetracycline MIC in µg/mL") + geom_bar(fi
ll = "#155547") + theme_pubclean() +
      scale_x_discrete(breaks=c("1","2"),labels=c("1 to 2", ">8"))
tia <- ggplot(ecoli.ordinal, aes(TIA)) + xlab("Tiamulin MIC in µg/mL") + geom_bar(fill =
"#ce6b11") + theme_pubclean() +
      scale_x_discrete(breaks=c("1","2"),labels=c("16 to 32", ">32"))
ggarrange(gen, neo, sul, spc, tet, tia, ncol = 3, nrow = 2)
```

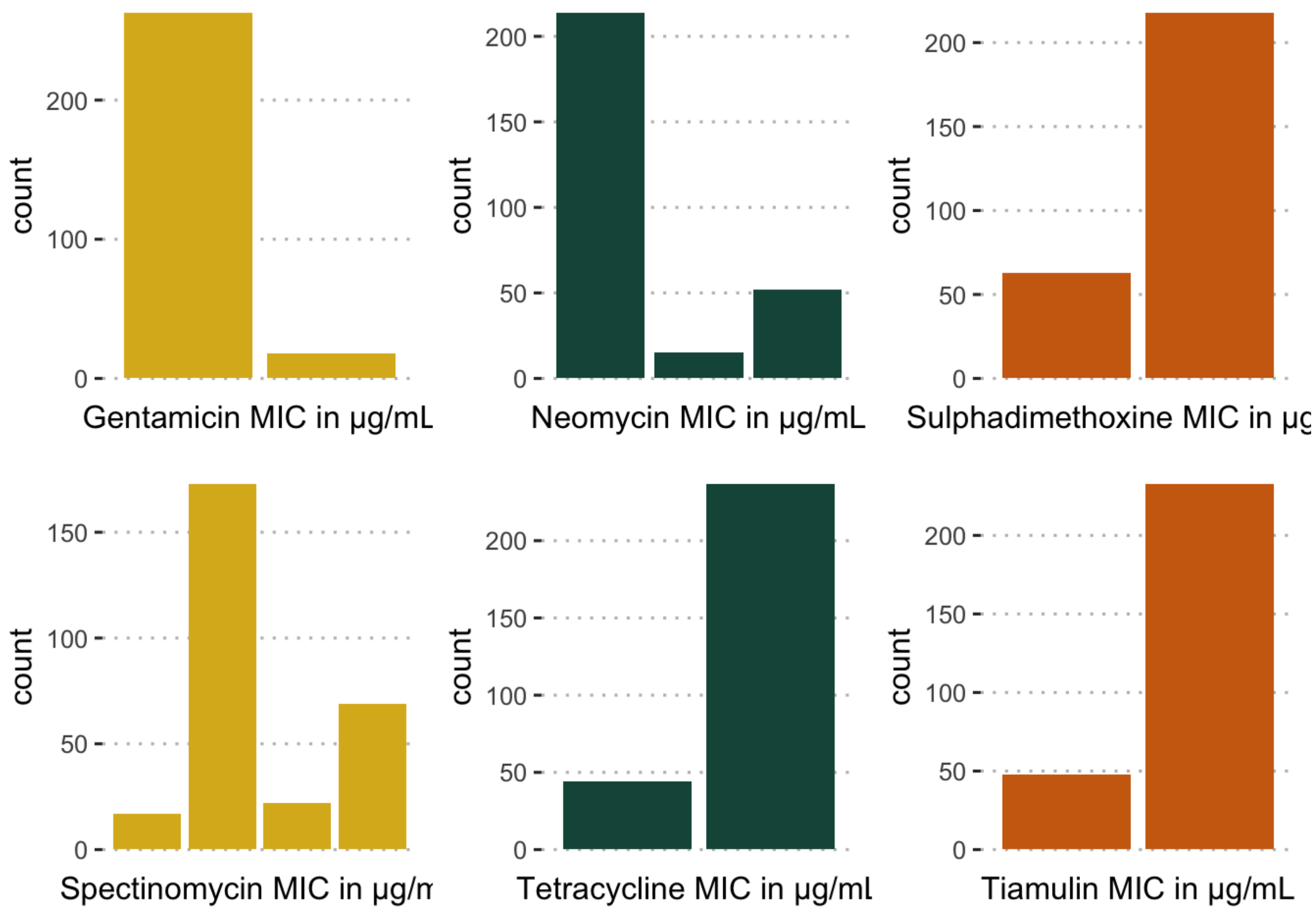


```
tild <- ggplot(ecoli.ordinal, aes(TILD)) + xlab("Tildipirosin MIC in µg/mL") + geom_bar
(fill = "#dbb522") + theme_pubclean() +
      scale_x_discrete(breaks=c("1","2","3"),labels=c("<=1 to 2", "4", "8 to >16"))
sxt <- ggplot(ecoli.ordinal, aes(SXT)) + xlab("Trimethoprin-sulfameth MIC in µg/mL") + g
eom_bar(fill = "#155547") + theme_pubclean() +
      scale_x_discrete(breaks=c("1","2"),labels=c("<=2", ">2"))
tul <- ggplot(ecoli.ordinal, aes(TUL)) + xlab("Tulathromycin MIC in µg/mL") + geom_bar(f
ill = "#ce6b11") + theme_pubclean() +
      scale_x_discrete(breaks=c("1","2"),labels=c("<=8", "16 to 64"))
ggarrange(tild, sxt, tul, ncol=3, nrow=2)
```

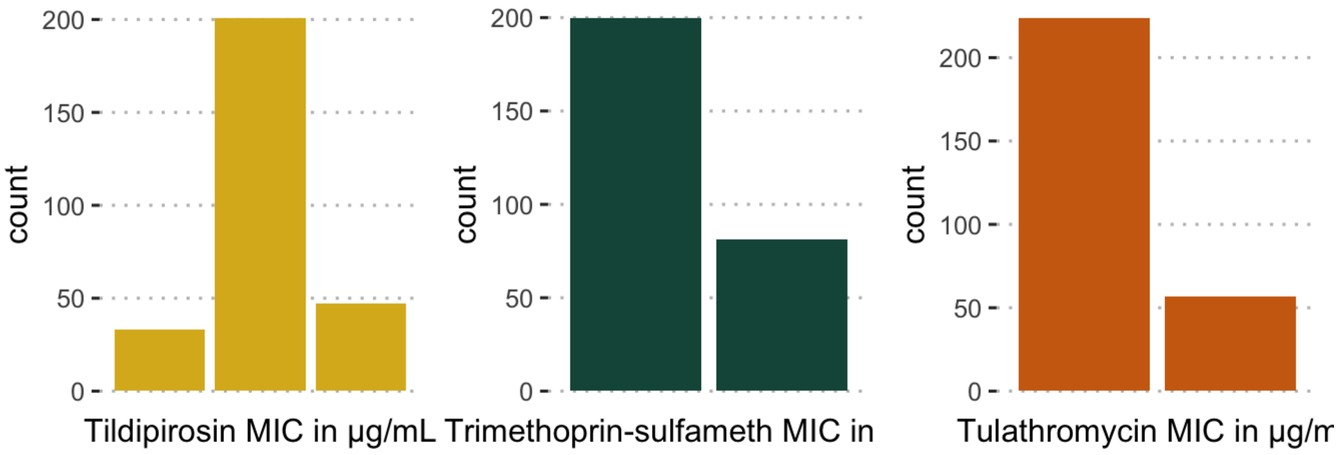

Learning the Bayesian Network

We use the Jonckheere-Terpstra test, a conditional independence test that should be used in bnlearn package for ordinal data (https://www.bnlearn.com (https://www.bnlearn.com)). It is a trend test and should only be used with constraint-based algorithms

We build graphs using the three constraint-based learning algorithms offered in bnlearn (pc, incremental association, and grow-shrink)

First, pc, a modern implementation of the first practical constraint-based structure learning algorithm

```
pc.graph <- pc.stable(ecoli.ordinal, cluster = NULL, whitelist = NULL, blacklist = NULL,
test = "jt", alpha = 0.05, B = NULL,
                      max.sx = NULL, debug = FALSE, undirected = TRUE)
graphviz.plot(pc.graph, shape = "ellipse")
```

```
## Loading required namespace: Rgraphviz
```

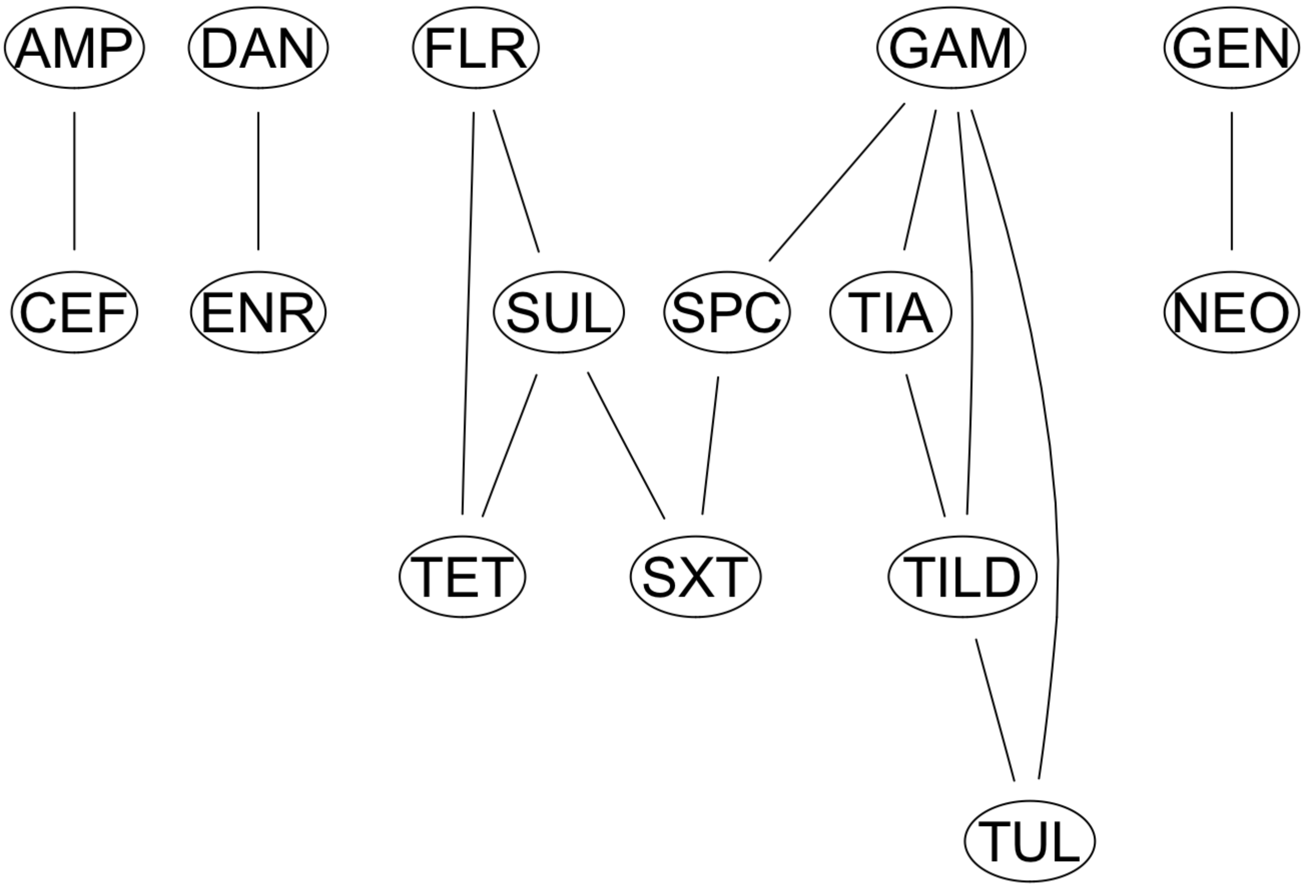

Second, iamb, based on the Markov blanket detection algorithm of the same name, a two-based selection scheme (forward selection followed by an attempt to remove false positives)

```
iamb.graph <- iamb(ecoli.ordinal, whitelist = NULL, blacklist = NULL, test = "jt", alpha
= 0.05, B = NULL,
                   max.sx = NULL, debug = FALSE, undirected = TRUE)
graphviz.plot(iamb.graph, shape = "ellipse")
```

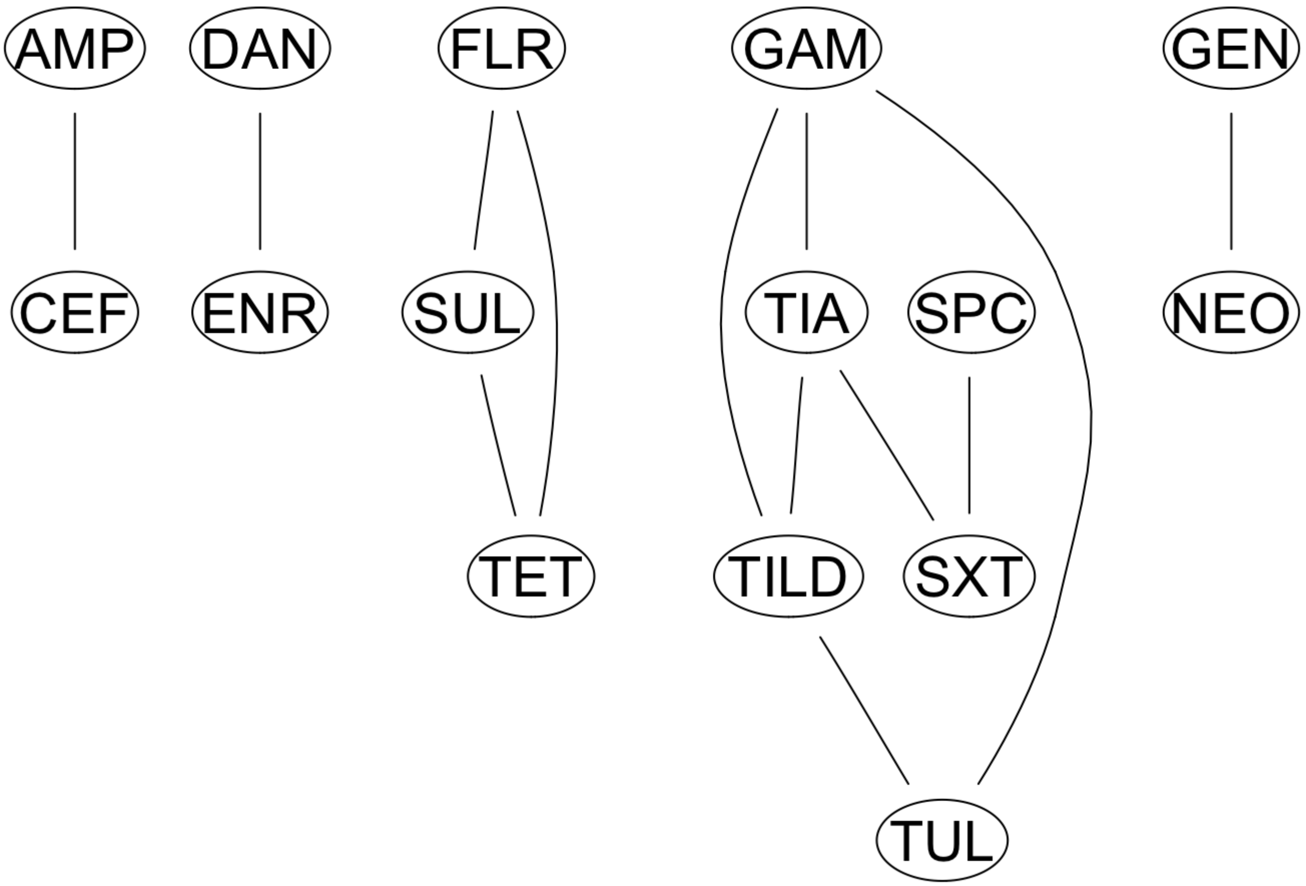

Finally, gs, based on the grow-shrink Markov blanket, the first and simplest Markov blanket detection algorithm used in a structure learning algorithm

```
gs.graph <- gs(ecoli.ordinal, whitelist = NULL, blacklist = NULL, test = "jt", alpha = 
0.05, B = NULL,
               max.sx = NULL, debug = FALSE, undirected = TRUE)
graphviz.plot(gs.graph, shape = "ellipse")
```

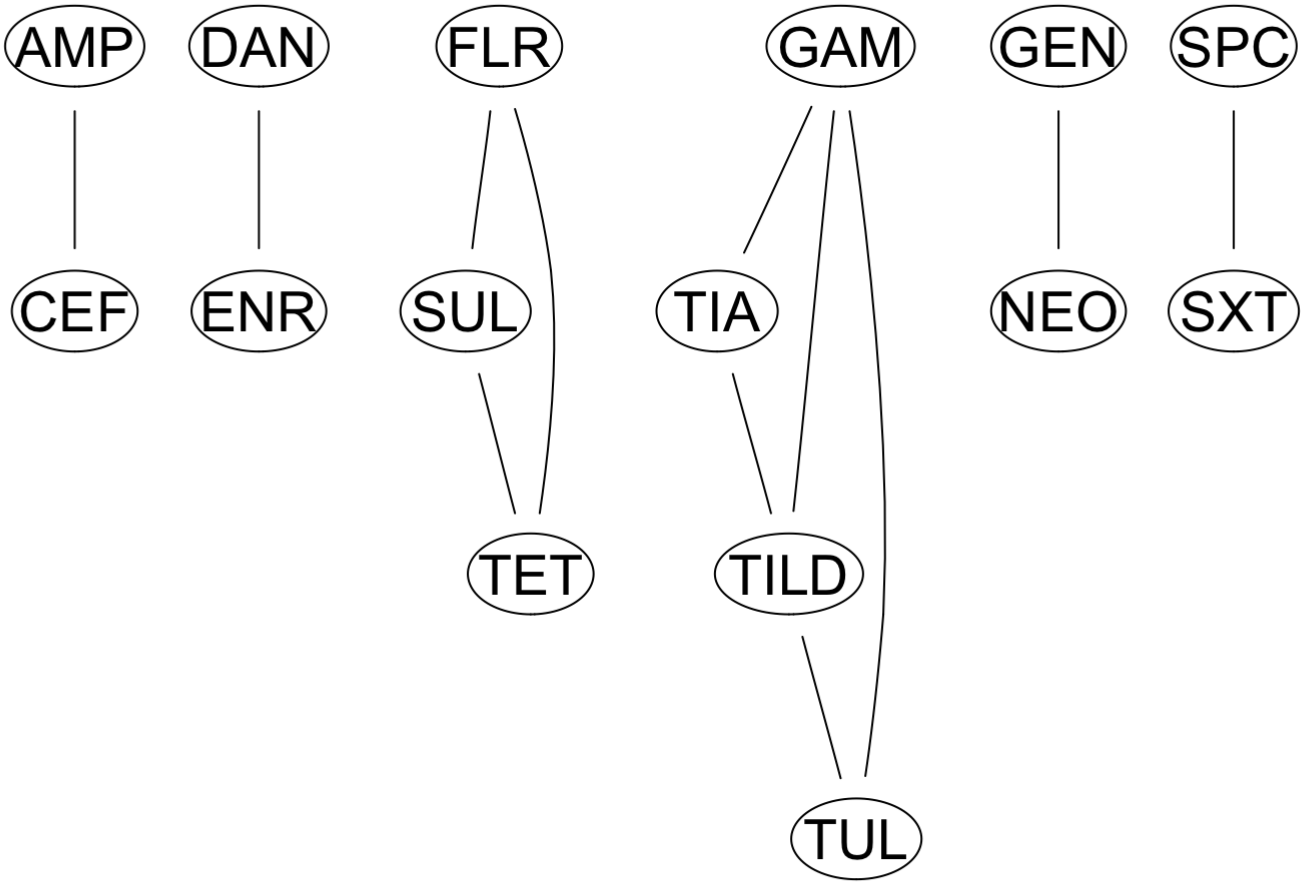

We can plot the graphs next side-by-side and in the same orientation to compare

```
par(mfrow = c(1,3))
graphviz.compare(pc.graph, iamb.graph, gs.graph, shape = "ellipse",
                 main = c("PC GRAPH", "IAMB GRAPH", "GS GRAPH"))
```

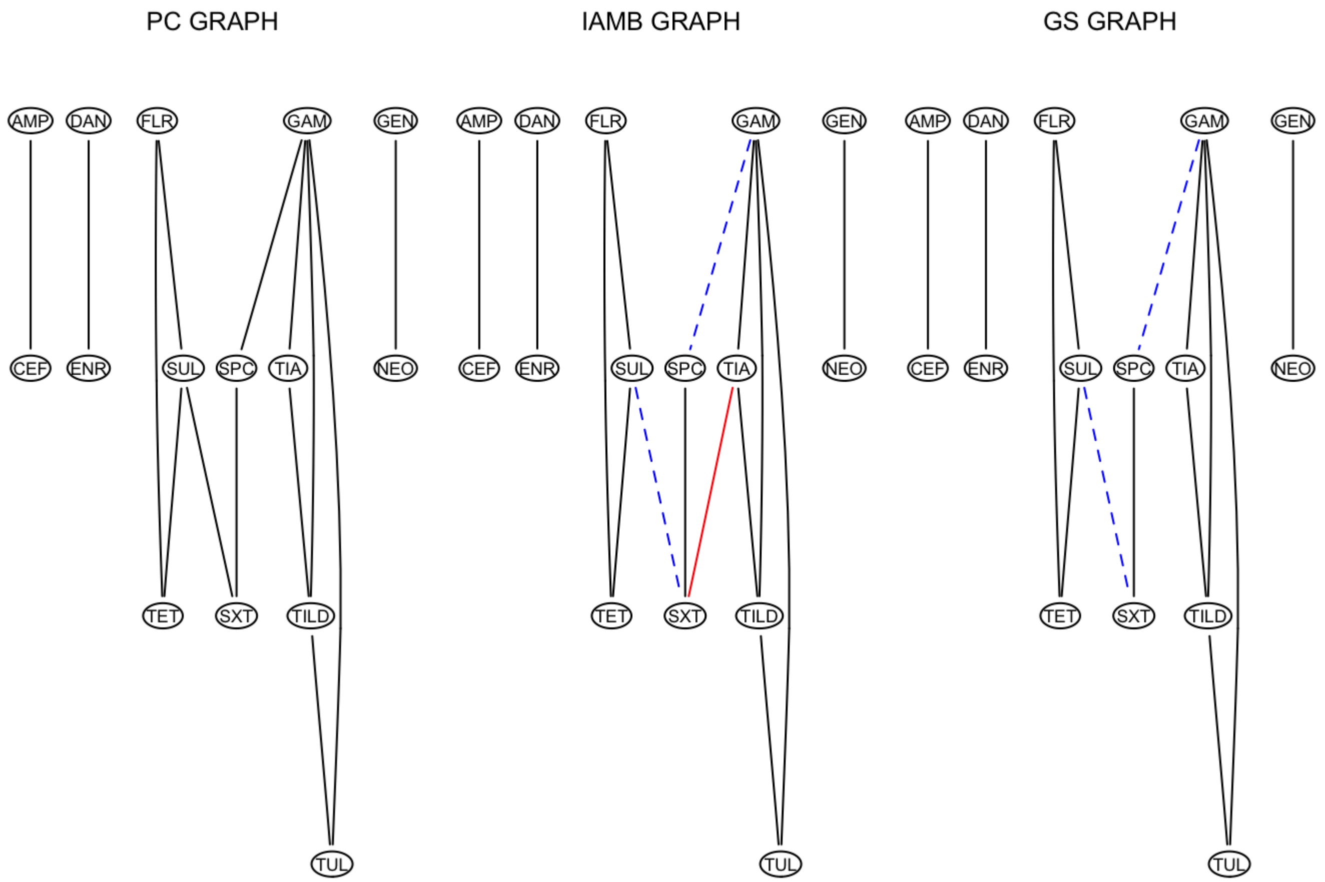


```
par(mfrow = c(1,1))
```

Bootstrap Aggregation

Model averaging helps obtain a stable network structure from the data. Using nonparametric bootstrap and learning one network from each bootstrap sample

```
boot = boot.strength(ecoli.ordinal, R = 10000, algorithm = "pc.stable",
                     algorithm.args = list(test = "jt"))

avg.boot = averaged.network(boot, threshold = 0.50)
graphviz.plot(avg.boot, shape = "ellipse")
```

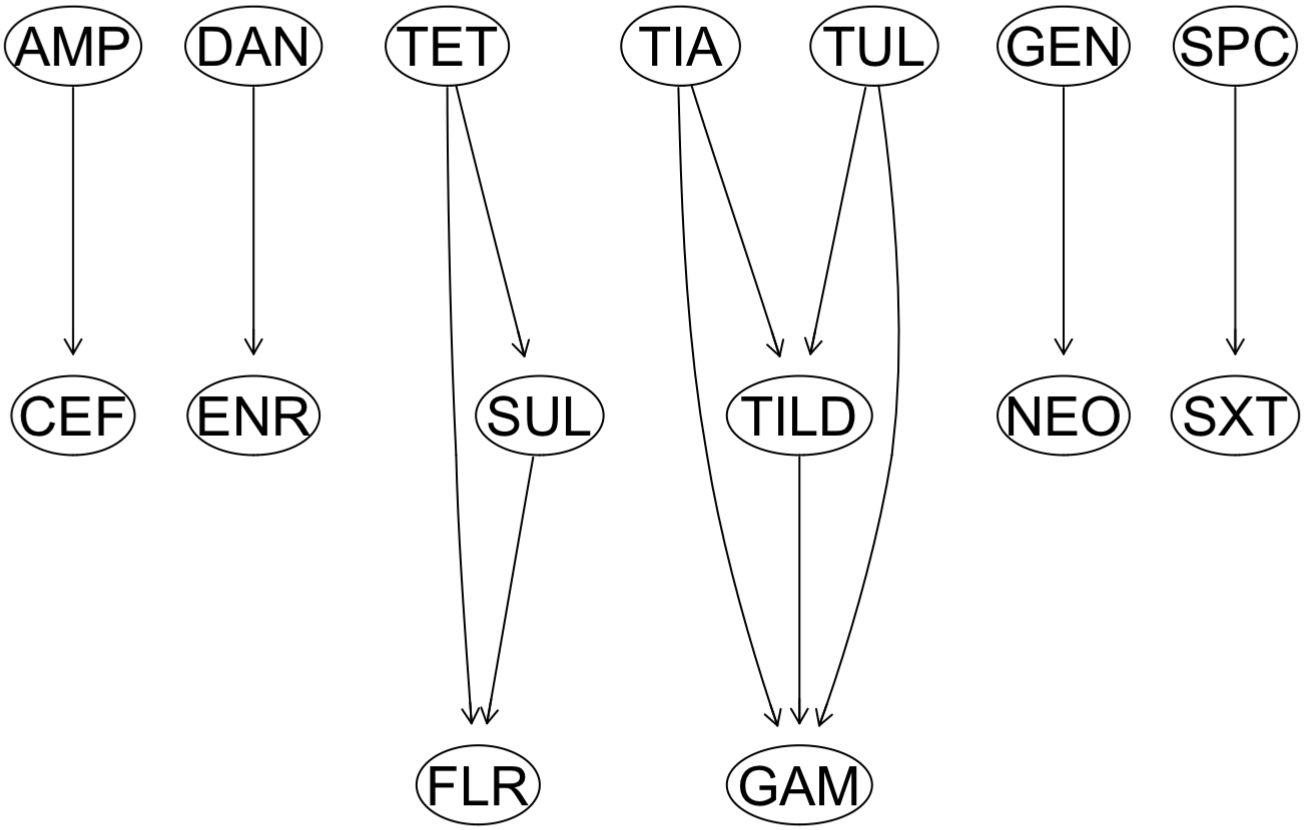

Investigate the threshold and compare with the threshold learned from the data

```
plot(boot)
```

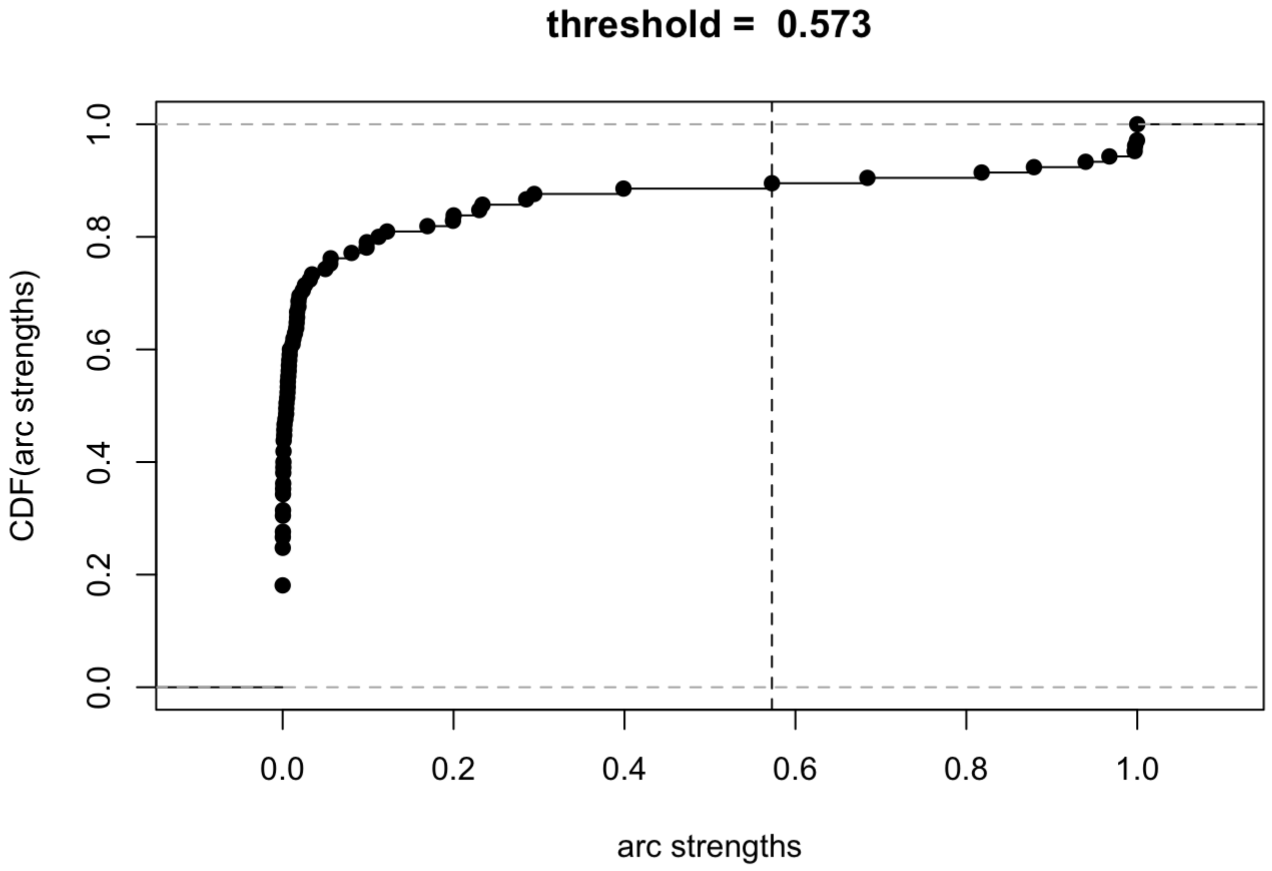

Plot strength graph where thickness of lines correspond with strength of association

```
strength.plot(avg.boot, boot, shape = "ellipse")
```

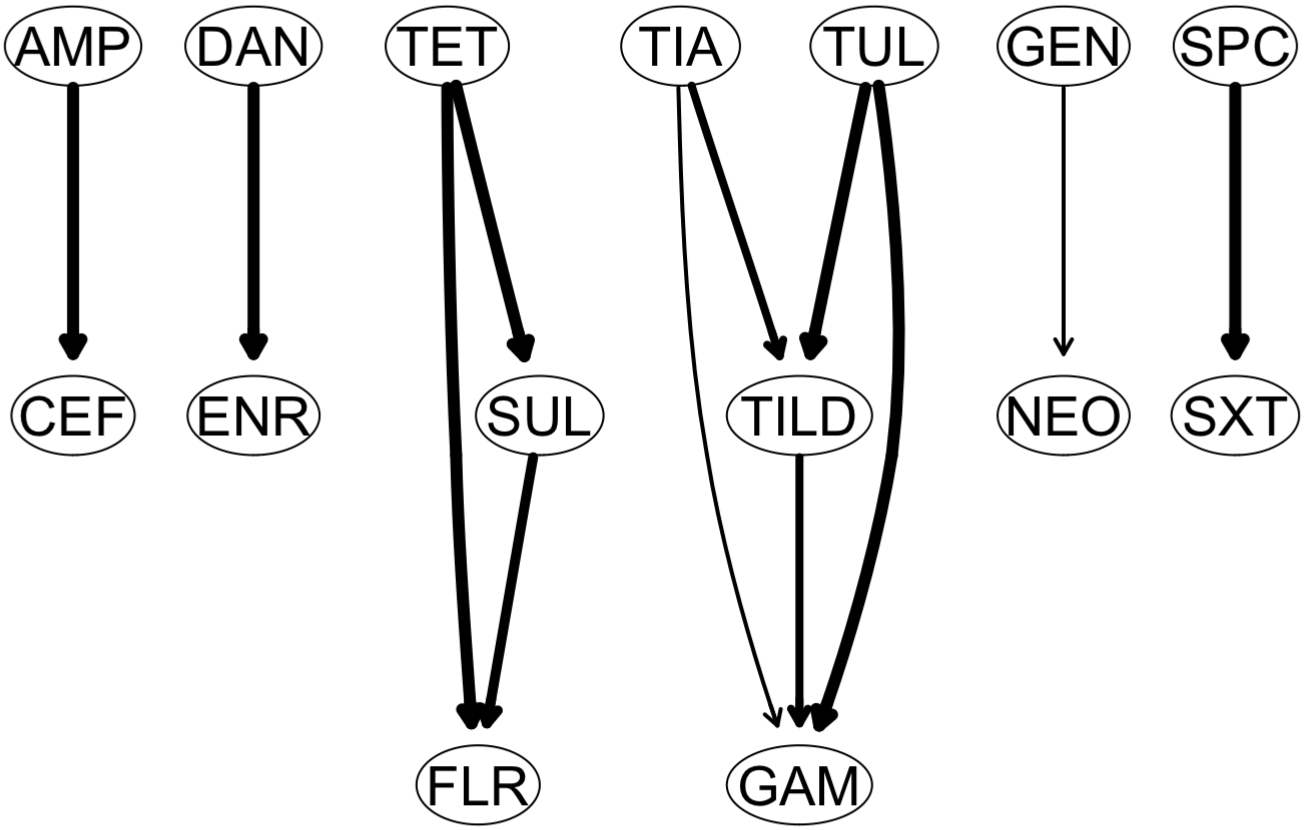

Compare all graphs built

```
par(mfrow = c(2,2))
graphviz.compare(pc.graph, iamb.graph, gs.graph, avg.boot, shape = "ellipse",
                 main = c("PC GRAPH", "IAMB GRAPH", "GS GRAPH", "AVERAGE PC GRAPH"))
```

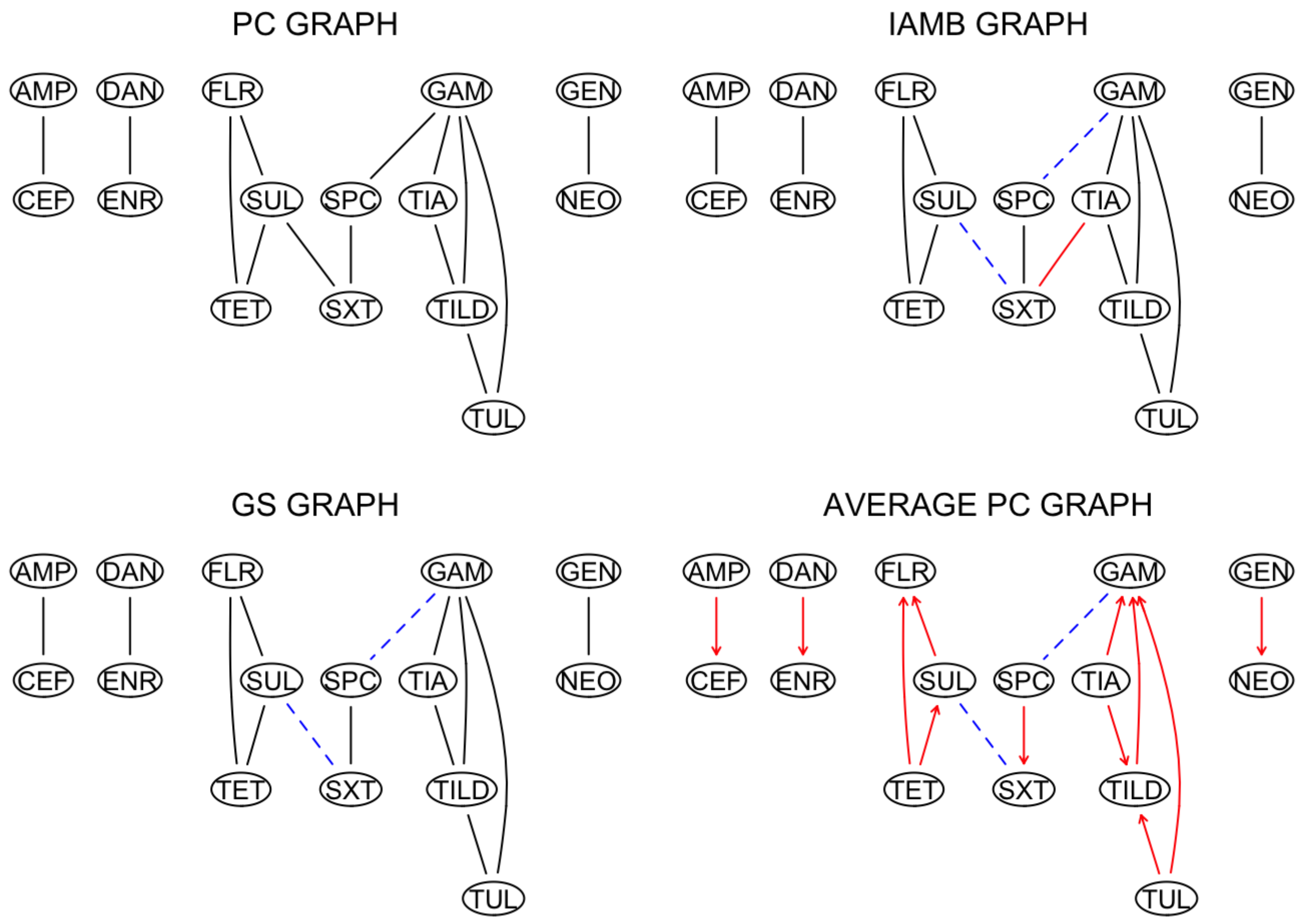


```
par(mfrow = c(1,1))
```

Parameter Learning

Graph must be directed to fit parameters, so will not suppress arc direction First, we will fit the parameters with the “bayes” method

```
avg.bayes = bn.fit(avg.boot, ecoli.ordinal, method = "bayes")
```

View the overall conditional probability table

```
avg.bayes
```

```
##
##   Bayesian network parameters
##
##   Parameters of node AMP (ordinal distribution)
##
## Conditional probability table:
##    0.5 to 1          2     4 to 5        >16
## 0.05053191 0.30939716 0.44060284 0.19946809
##
##   Parameters of node CEF (ordinal distribution)
##
## Conditional probability table:
##
##           AMP
## CEF            0.5 to 1            2       4 to 5          >16
##   <=0.25   0.7061403509 0.6654727794 0.2097585513 0.0900000000
##   0.5 to 4 0.2850877193 0.3330945559 0.7892354125 0.3922222222
##   8        0.0043859649 0.0007163324 0.0005030181 0.2677777778
##   >8       0.0043859649 0.0007163324 0.0005030181 0.2500000000
##
##   Parameters of node DAN (ordinal distribution)
##
## Conditional probability table:
##      <=0.12       0.25   0.5 to 1         >1
## 0.77748227 0.04698582 0.07535461 0.10017730
##
##   Parameters of node ENR (ordinal distribution)
##
## Conditional probability table:
##
##           DAN
## ENR              <=0.12         0.25     0.5 to 1           >1
##   <=0.12   0.9991448119 0.0801886792 0.0029411765 0.0022123894
##   0.25     0.0002850627 0.9103773585 0.5205882353 0.0022123894
##   0.5 to 1 0.0002850627 0.0047169811 0.4735294118 0.2853982301
##   >1       0.0002850627 0.0047169811 0.0029411765 0.7101769912
##
##   Parameters of node FLR (ordinal distribution)
##
## Conditional probability table:
##
## , , TET = 1 to 2
##
##         SUL
## FLR             <=256         >256
##   1 to 2 0.3250517598 0.4901960784
##   4 to 8 0.6728778468 0.2549019608
##   >8     0.0020703934 0.2549019608
##
## , , TET = >8
##
##         SUL
## FLR             <=256         >256
```

```
##   1 to 2 0.0465949821 0.0003889537
##   4 to 8 0.4336917563 0.1030727343
##   >8     0.5197132616 0.8965383119
##
##
##   Parameters of node GAM (ordinal distribution)
##
## Conditional probability table:
##
## , , TILD = <=1 to 2, TUL = <=8
##
##           TIA
## GAM            16 to 32          >32
##   <=1 to 4 0.8310291859 0.6643678161
##   8        0.0568356375 0.1678160920
##   >8       0.1121351767 0.1678160920
##
## , , TILD = 4, TUL = <=8
##
##           TIA
## GAM            16 to 32          >32
##   <=1 to 4 0.5179487179 0.2567998499
##   8        0.4810256410 0.6619771150
##   >8       0.0010256410 0.0812230351
##
## , , TILD = 8 to >16, TUL = <=8
##
##           TIA
## GAM            16 to 32          >32
##   <=1 to 4 0.0256410256 0.0015360983
##   8        0.9487179487 0.8310291859
##   >8       0.0256410256 0.1674347158
##
## , , TILD = <=1 to 2, TUL = 16 to 64
##
##           TIA
## GAM            16 to 32          >32
##   <=1 to 4 0.0256410256 0.0133333333
##   8        0.0256410256 0.0133333333
##   >8       0.9487179487 0.9733333333
##
## , , TILD = 4, TUL = 16 to 64
##
##           TIA
## GAM            16 to 32          >32
##   <=1 to 4 0.0256410256 0.0011074197
##   8        0.0256410256 0.5592469546
##   >8       0.9487179487 0.4396456257
##
## , , TILD = 8 to >16, TUL = 16 to 64
##
##           TIA
## GAM            16 to 32          >32
##   <=1 to 4 0.3333333333 0.0009891197
```

```
##   8         0.3333333333 0.6419386746
##   >8        0.3333333333 0.3570722057
##
##
##   Parameters of node GEN (ordinal distribution)
##
## Conditional probability table:
##         <=1   2 to >16
## 0.93439716 0.06560284
##
##   Parameters of node NEO (ordinal distribution)
##
## Conditional probability table:
##
##            GEN
## NEO                 <=1   2 to >16
##   <=4 to 16 0.79000633 0.33333333
##   32        0.05376344 0.06306306
##   >32       0.15623023 0.60360360
##
##   Parameters of node SUL (ordinal distribution)
##
## Conditional probability table:
##
##        TET
## SUL         1 to 2         >8
##   <=256 0.90449438 0.09789474
##   >256  0.09550562 0.90210526
##
##   Parameters of node SPC (ordinal distribution)
##
## Conditional probability table:
##         <=8         16   32 to 64        >64
## 0.06117021 0.61436170 0.07890071 0.24556738
##
##   Parameters of node TET (ordinal distribution)
##
## Conditional probability table:
##     1 to 2        >8
## 0.1578014 0.8421986
##
##   Parameters of node TIA (ordinal distribution)
##
## Conditional probability table:
##   16 to 32       >32
## 0.1719858 0.8280142
##
##   Parameters of node TILD (ordinal distribution)
##
## Conditional probability table:
##
## , , TUL = <=8
##
##           TIA
```

```
## TILD         16 to 32        >32
##   <=1 to 2 0.39099099 0.06778869
##   4        0.58558559 0.83076204
##   8 to >16 0.02342342 0.10144928
##
## , , TUL = 16 to 64
##
##           TIA
## TILD         16 to 32        >32
##   <=1 to 2 0.48148148 0.03770739
##   4        0.48148148 0.45399698
##   8 to >16 0.03703704 0.50829563
##
##
##   Parameters of node SXT (ordinal distribution)
##
## Conditional probability table:
##
##      SPC
## SXT           <=8          16    32 to 64         >64
##   <=2 0.992753623 0.935786436 0.365168539 0.189530686
##   >2  0.007246377 0.064213564 0.634831461 0.810469314
##
##   Parameters of node TUL (ordinal distribution)
##
## Conditional probability table:
##        <=8  16 to 64
## 0.7960993 0.2039007
```

Query the graph for specific probabilities P(AMP | CEF)

```
cpquery(avg.bayes, (AMP == ">16"), CEF == ">8")
```

```
## [1] 0.9823183
```

```
cpquery(avg.bayes, (AMP == ">16"), CEF == "8")
```

```
## [1] 0.987061
```

```
cpquery(avg.bayes, (AMP == ">16"), CEF == "0.5 to 4")
```

```
## [1] 0.1401578
```

```
cpquery(avg.bayes, (AMP == ">16"), CEF == "<=0.25")
```

```
## [1] 0.05085714
```

```
cpquery(avg.bayes, (AMP == "0.5 to 1"), CEF == "<=0.25")
```

```
## [1] 0.09513151
```

P(CEF | AMP)

```
cpquery(avg.bayes, (CEF == ">8"), AMP == ">16")
```

```
## [1] 0.25
```

```
cpquery(avg.bayes, (CEF == "8"), AMP == ">16")
```

```
## [1] 0.2623788
```

```
cpquery(avg.bayes, (CEF == "0.5 TO 4"), AMP == ">16")
```

```
## [1] 0
```

```
cpquery(avg.bayes, (CEF == "<=0.25"), AMP == ">16")
```

```
## [1] 0.09346272
```

```
cpquery(avg.bayes, (CEF == ">8"), AMP == "0.5 to 1")
```

```
## [1] 0.003861004
```

```
cpquery(avg.bayes, (CEF == "<=0.25"), AMP == "0.5 to 1")
```

```
## [1] 0.7080745
```

P(TET | SUL & FLR)

```
cpquery(avg.bayes, (TET == ">8"), SUL == ">256" & FLR == "1 to 2")
```

```
## [1] 0.03092784
```

```
cpquery(avg.bayes, (TET == ">8"), SUL == "<=256" & FLR == "1 to 2")
```

```
## [1] 0.09411765
```

```
cpquery(avg.bayes, (TET == ">8"), SUL == ">256" & FLR == ">8")
```

```
## [1] 0.9934172
```

```
cpquery(avg.bayes, (TET == ">8"), SUL == "<=256" & FLR == ">8")
```

```
## [1] 0.9951691
```

```
cpquery(avg.bayes, (TET == "1 to 2"), SUL == ">256" & FLR == "1 to 2")
```

```
## [1] 0.9230769
```

```
cpquery(avg.bayes, (TET == "1 to 2"), SUL == "<=256" & FLR == "1 to 2")
```

```
## [1] 0.9084249
```

```
cpquery(avg.bayes, (TET == "1 to 2"), SUL == ">256" & FLR == ">8")
```

```
## [1] 0.006213873
```

```
cpquery(avg.bayes, (TET == "1 to 2"), SUL == "<=256" & FLR == ">8")
```

```
## [1] 0.006622517
```

This format can be followed to query any connections in the graph
